# Influence of fractional carbon-dioxide laser in comparison to ErCr-YSGG on the dentin bond integrity of bioactive materials

**DOI:** 10.12669/pjms.36.3.1819

**Published:** 2020

**Authors:** Zaid Al-Jeaidi

**Affiliations:** 1 Zaid Al-Jeaidi Associate Professor, Conservative Dental Science Department, College of Dentistry, Prince Sattam Bin Abdulaziz University, Al-Kharj, Saudi Arabia

**Keywords:** Fractional carbon dioxide laser, Er-Cr-YSGG laser, Bioactive material, Dentin bonding, Microleakage

## Abstract

**Objective:**

The aim was to assess the influence of Er, Cr: YSGG laser (ECL) and fractional carbon dioxide laser (FCL) on the shear bond strength (SBS) and microleakage of bioactive restorative material to dentin.

**Methods:**

The study was performed in King Saud university in the month of June-July 2019. One hundred and twenty permanent teeth were vertically placed in acrylic resin. Based on the type of surface treatment regime (n=40), samples were divided into three groups. Group-I samples were surface conditioned with total etch and rinse (TE); Group-2 samples were surface treated with Er, Cr: YSGG laser (ECL) and Group-3 specimens were conditioned with fractional carbon dioxide laser (FCL). Surface treatment of dentin was followed by type of bulk fill resin (BFR) application. Tetric-N-Ceram was bonded to dentin conditioned with TE (n=20), FCL (n=20) and ECL (n=20). Similarly, bioactive material (BAM) was also bonded to conditioned surface (n=60). Samples (n=10) among each group were placed in a Universal testing machine. For microleakage testing 5 pairs of samples from each group (n=10) were placed in solution of 2% methylene blue for 24h Fracture analysis was performed using stereomicroscope at 40x magnification. Descriptive statistics i.e., means and standard for SBS and microleakage were compared using analysis of variance (ANOVA) and Tukey’s post hoc test at a significance level of (p < 0.05)

**Results:**

The highest SBS scores were displayed by TE-BFR (Bulk filled resin) (19.21 ± 0.925 Mpa) and the lowest shear bond scores were presented by FCL-BFR (11.06±1.611 Mpa). The lowest microleakage scores were exhibited by group ECL-BFR (24.11±13.01nm). Similarly, the highest microleakage score was displayed in group FCL-BAM (42.18±16.32 nm). Admixed failure was pertinent in groups conditioned by ECL. Moreover, groups conditioned with FCL adhesive type of failure was found in abundance.

**Conclusions:**

ECL has a potential to be used as an alternate to total etch and rinse for conditioning of dentin when bonded to bioactive materials.

## INTRODUCTION

The success of the restorative material is dependent on excellent adhesion and reasonable sealing of the cavity walls.^1^ To find a material with such characteristic, led to the development of resin bulk fill material.^1^,^2^ Bulk fill resin composites were introduced to reduce technique sensitivity by reducing placement time and promoting single phase cure up to the depth of 4mm.^3^ Moreover, different types of polymerization techniques (self-cure, light cure and dual cure) with diverse physical properties makes bulk fill composites an ideal choice in load bearing areas and as an aesthetic restoration.^4^

Recent, advancement in restorative dentistry has led to the development of bioactive material (BAM) which directly links and recharges itself to changes in oral cavity.^5^ BAM is composed of glass particles and resin matrix which facilities diffusion of calcium, phosphate and fluoride ions and neutralizes the oral pH, remineralizes the dentin, exhibits antimicrobial activity and forms a chemical bond improving marginal adaptability decreasing microleakage.^5^

Microleakage defines the success of a treatment outcome and is one of the contributing factors responsible for secondary caries and pulpal irritation leading to poor dental prognosis.^6^ In order to minimize microleakage and make bonding to dentin more predictable, innovative procedures including the use of lasers i.e., Er,Cr:YSGG laser (ECL) and fractional carbon dioxide laser (CO_2_), for enamel and lithium disilicate (LD) conditioning has exhibited evidence based outcomes.^7^,^8^ Both these laser treatments (in-vitro) have gained popularity due to their predictable results, safety and low technique sensitivity compared to conventional dentin treatment methods.^9^

To our knowledge from indexed literature, evidence related to CO_2_ laser used as a dentin conditioner is scarce and available evidence on contemporary developed lasers is controversial.^10^ Moreover, ECL effects on dentin surface has demonstrated convincing outcomes 9,11 but there are no studies to compare dentin conditioned with ECL and CO_2_ when bonded to BAM. It is hypothesized that dentin conditioned with CO_2_ and ECL and bonded to BAM will show comparable outcome to dentin treated with conventional conditioning method when bonded to BAM. Therefore, the aim of the present study was to assess shear bond strength (SBS) and microleakage scores (MS) of dentin conditioned with CO_2_ and ECL when bonded to conventional bulk fill resin and bioactive restorative material.

## METHODS

One hundred and twenty permanent non-carious, intact, non-fractured permanent third molars were isolated and cleaned from debris and inorganic remnants with the help of periodontal curette and scaler (Superior Instruments Co, New York, USA). The teeth were stored in thymol solution (0.1%) to disinfect for two weeks and stored in distilled water at 4**°**C. Within the sections of polyvinyl pipes (10mm diameter) teeth were vertically placed in acrylic resin (Ortho-Jet, Lang Dental MFG, IL, USA) up to the cementoenamel junction. To maintain uniformity, the dentin surface was polished with silicon carbide paper (Buehler, Lake Bluff, IL, USA) under water irrigation for 10 sec at 250rpm using a polishing machine (Aropol 2V, Arotec). All specimen were bathed in ultra-sonic solution (Luxor Clean, USA) for 60 sec. The study was performed in King Saud university in June-July 2019. The study followed the Check List for Reporting in Vitro Study (CRIS) guidelines.

### Dentine Conditioning Regime

Based on the type of surface treatment regime (n=40), samples were divided into three groups. Group-I samples were surface conditioned with total etch and rinse (TE); Group-2 samples were surface treated with Er,Cr:YSGG laser (ECL) and Group-3 specimens were conditioned with fractional carbon dioxide laser (FCL). For the surface treatment the following protocol was followed:

### Group-I (TE)

The dentin surface was conditioned using 35% phosphoric acid (Ultra-Etch; Ultradent Products, Inc., South Jordan, UT, USA) for 15sec and rinsed for 10sec. A universal bonding agent (Tetric N-Bond Universal, Ivoclar-Vivadent) was agitated and light cured (Bluephase G2, Ivoclar, Vivadent) for 20 s on 40 specimens only.

### Group-2 (ECL)

Buccal dentinal surface of each specimen was conditioned by ECL (Waterlase C-100, BioLase Tech Inc., California, USA) power 4.5W and frequency 30Hz in a non-contact mode from 2mm using tip (MZ=10) for a duration of 60 seconds.

### Group-3 (FCL)

FCL in a contact mode perpendicular to the dentinal surface at 0.8W power and 10Hz frequency was irradiated for 60 sec.

Surface treatment of dentin was followed by type of bulk fill resin (BFR) application. Tetric-N-Ceram (Ivoclar, Vivadent) was bonded to dentin conditioned with TE (n=20), FCL (n=20) and ECL (n=20). Similarly, bioactive material (BAM) (Activa, Pulpdent Cooperation, Watertown, Massachusetts USA) was also bonded to conditioned surface (n=60). The bonding of bulk fill application was done in accordance to manufacturer recommendation. This resulted in a total of six groups each for shear bind strength and microleakage testing with 10 specimens in each (n=120). All specimens were stored in distilled water at room temperature for 24hrs and then thermocycled between 5C to 55C for 10000 cycles to simulate oral conditions.

### Shear Bond Strength (SBS) testing

Specimen (n=10) among each group were placed in a Universal testing machine (Instron Santam, model STM-20, Riyadh, KSA) under a cross head speed of 0.5mm/ min using a 1-mm diameter metallic plunger. The force was applied parallel to the bonded surfaces. The SBS was calculated in mega pascal (MPa).

### Microleakage testing (MT)

For microleakage testing five pairs of sample from each group (n=10) were placed in solution of 2% methylene blue for 24 hour and then washed under running water. Specimens were sectioned in a longitudinal direction using low-speed carbon discs (3M ESPE Dental Products, St. Paul, MN, USA) under water spray. Each longitudinal section was observed in an image analysis software (DP soft, Olympus Optical, Middlesex) on a digital microscope (Hirox- KH7700).

### Failure mode Analysis

Fracture analysis was performed by two examiners using stereomicroscope at 40x magnification (SZX7, Olympus, Hamburg, Germany). Type of failure was classified into adhesive (failure in bonded interface), cohesive (failure in bonded material or dentin) and mixed (combination of both adhesive and cohesive)

### Statistical Analysis

BS and microleakage data was tabulated using statistical program for social science (SPSS version 19, Inc., Chicago, US). Normality of data obtained was assessed using Kolmogorov-Smirnov test. Descriptive statistics i.e., means and standard for SBS and microleakage were compared using analysis of variance (ANOVA) and Tukey’s post hoc test at a significance level of (p < 0.05).

## RESULTS

### SBS scores

The highest SBS scores were displayed by TE-BFR (Bulk filled resin) (19.21 ± 0.925 Mpa) and the lowest shear bond scores were presented by FCL-BFR (11.06±1.611 Mpa). SBS were comparable among experimental groups FCL-BFR (11.06 ± 1.611) and FCL-BAM (13.45 ± 2.459 Mpa) (p>0.05). Similarly, no statistical difference was observed in groups conditioned with TE and ECL and bonded to bulk fill material BAM and BFR (p>0.05). For bond strength values, analysis of variance (ANOVA) showed significant difference among study groups p<0.001. (Table-I)

**Table-I:** Descriptive statistics of SBS in MPa among experimental groups using ANOVA and Tukey multiple comparisons test.

Surface conditioning/ Type of bulk fill material	Mean (MPa) ± SD (MPa)	p value!
TE-BFR†(Control)	19.21 ± 0.925	<0.001
TE-BAM †(Control)	17.24 ± 1.123	
ECL-BFR †	16.13 ± 3.012	
ECL-BAM†	16.25 ± 2.102	
FCL-BFR ¶	11.06 ± 1.611	
FCL-BAM ¶	13.45 ± 2.459	

**TE:** Total Etch, BFR: Tetric-N-Ceram, **FCL:** Fractional carbon dioxide laser,

**ECL:** Er, Cr: YSGG, **BAM:** Bioactive material.

The highest and lowest SBS values are in bold

† Significantly different from groups- FCL-BFR, FCL-BAM (p <0.05)

¶ Significantly different from groups- TE-BFR, TE-BAM, ECL-BFR, ECL-BAM

(Tukey multiple comparison test)

! Showing significant difference among study group (ANOVA).

### Microleakage outcome

The lowest microleakage scores were exhibited by group ECL-BFR (24.11±13.01nm). Similarly, the highest microleakage score was displayed in group FCL-BAM (42.18±16.32nm). Microleakage scores of samples in group TE-BAM (37.55±12.551nm), ECL-BAM (41.08±14.78) and FCL-BAM (42.18±16.325) presented comparable outcomes (p>0.05). For microleakage values, analysis of variance (ANOVA) showed significant difference among the study groups (p<0.001). Moreover, bulk fill BAM (Activa) when bonded to different surface treatment regimes showed no significant difference among groups (p>0.05). (Table-II)

**Table-II:** Descriptive statistics of microleakage scores among study groups using ANOVA and Tukey multiple comparisons test.

Surface conditioning/ Nature of bulk fill material	Mean (nm)	SD (nm)	P value!
TE-BFR* (Control)	28.11	13.657	<0.001
TE-BAM †(Control)	37.55	12.551	
ECL-BFR *	24.11	13.011	
ECL-BAM†	41.08	14.784	
FCL-BFR *	33.98	14.855	
FCL-BAM †	42.18	16.325	

**TE:** Total Etch, **BFR:** Tetric-N-Ceram, **FCL:** Fractional carbon dioxide laser,

**ECL:** Er, Cr: YSGG, BAM: Bioactive material, The highest and lowest microleakage values are in bold

† Significantly different from groups TE-BFR, ECL-BFR, FCL-BFR (p <0.05)

* Significantly different from groups- TE-BAM, ECL-BAM, FCL-BAM (p <0.05)

(Tukey multiple comparison test)

! Showing significant difference among study group (ANOVA).

### Failure mode

In samples conditioned with TE, majority of the failures reported was cohesive. Whereas, admixed failure was pertinent in groups conditioned by ECL. Moreover, groups conditioned with FCL adhesive type of failure was found in abundance. (Table-IIII)

**Table-III:** Modes of failure among different experimental groups.

Experimental groups	Adhesive (%)	Cohesive (%)	Admixed (%)	n = 10
TE-BFR (Control)	25	55	20	10
TE-BAM (Control)	10	67	23	10
ECL-BFR	10	30	60	10
ECL-BAM	10	20	70	10
FCL-BFR	65	15	20	10
FCL-BAM	70	10	20	10

## DISCUSSION

The present study was based on the hypothesis that dentin conditioned with CO_2_ and ECL when bonded to BAM will show comparable SBS values to dentin treated with conventional conditioning method and bonded to composite resin. Interestingly, the hypothesis was accepted partly as dentin conditioned with ECL exhibited comparable outcome to conventional dentin treatment, bonded to BAM and resin composite. However, dentin conditioned with CO_2_ laser and bonded to BAM and BFR exhibited significantly less bond strength. Moreover, specimens treated with CO_2_ and ECL displayed microleakage scores comparable to controls.

In the current study, SBS was measured using universal testing machine. The method is found to be reliable, simple and gives comparative analysis between different groups. The test is widely used as no further processing of specimens are required after bonding procedure.^12^ In the present study, dentin conditioned with etch and rinse exhibited comparable outcome to dentin treated with ECL. Primary reason for this outcome is ECL at 2780nm is well absorbed by the dentin structure which enables expansion of dentinal tubules and disposal of organic and in-organic tissue producing irregular rugged appearance free from smear layer.^13^ Moreover, Tetric-N-bond (bulk fill resin) containing ethanol based solvent with low hydrophilicity and concentration gives better penetration into dentin conditioned by ECL improving bond strength values.^14^ In addition bonding of BAM to dentin is due to hydrophilic nature of BAM (Activa) creating a layer of ionic exchange matrix resulting in intimate adaption with tooth structure hence improving bond scores.^5^,^15^ The results of the present study are found to be in concurrence with the findings of Alkhudhairy et al.^16^ Moreover, the power and frequency (4.5W 30Hz) used in the existing study were similar to studies by Vohra et al.^9^, Alkhudhairy et al.^16^ and Chou et al.^17^ all proclaiming that dentin becomes more receptive and improves bond integrity at these parameters when conditioned with ECL.

Dentin conditioned with FCL exhibited low bond strength values compared to dentin conditioned with ECL and TE. There are no studies done to extrapolate or compare the findings of the present study. In the authors opinion low bond strength scores in FCL can be attributed to different laser parameters used in the present study. Moreover, at these laser settings FCL might have burned, vaporized and decreased dentin permeability compromising the substrate (dentin) integrity. This hypothesis needs validation and further studies should be performed using different laser parameters along with surface profilometry of lased dentin with FCL. In addition, the surface topography, surface roughness and strength of dentin after FCL conditioning should also be explored in a standardized environment.

To improve the longevity of restoration marginal integrity plays a crucial role. Integrity of any restoration is compromised by polymerizarion shrinkage.^18^ Magnitude of stress, C-factor and curing method are some factors causing untoward effects of polymerization shrinkage of restorative material.^19^ In the present study marginal integrity of restorative material was measured using a dye penetration test. The test is simple, in-expensive and convenient to use. Moreover, the dye has low molecular weight than bacteria and may detect penetration where bacterial penetration becomes impossible.^18^ The results of the present study indicate that bulk fill composite with Tetric-N-Ceram demonstrated the lowest microleakage scores. Manufacturers of Tetric N-Ceram claim that one layer placement up to 4mm, presence of Ivocerin as a novel light initiator, stress relievers and a low molecular weight mixture of bisphenol-A diglycidyldimethacrylate, UDMA, and ethoxylated bisphenol all are responsible for low microleakage scores.^20^ These findings of the present study are in agreement with Moosavi et al.^20^ and Zhu et al.^21^ Moreover, BAM (ACTIVA) demonstrated high microleakage scores in all experimental groups. This finding is in line with work by Owens et al.^5^ who demonstrated BAM (Activa) to have low microleakage at enamel margins compared to dentin. In the authors opinions, these results indicate that surface morphology of tooth plays a more significant role in microleakage scores compared to type of bulk fill material used for restorative procedure. More longitudinal clinical studies should be performed on BAM (Activa) to validate their clinical performance and efficiency.

Specimens lased with ECL demonstrated admixed type of failure. Thermo mechanical impact of ECL on dentinal surface, lateral forces, debonding protocol and nature of conditioning pattern may attribute this type of failure pattern.^22^ Moreover, adhesive failures were prevalent in samples conditioned with FCL. This failure type corresponds to low bond scores in FCL groups. Though, some authors found this type of failure favourable as there is less adhesive to remove from tooth surface but overall the nature of adhesive failure is conflicting.^23^,^24^

## CONCLUSION

Er,Cr:YSGG laser has a potential to be used as an alternate to total etch and rinse for conditioning of dentin when bonded to bioactive materials. Moreover, the conditioning of bonding surfaces and not material technologies play a significant role in microleakage of bioactive materials.
